# Novel *TBX20* Variations Susceptible to Sporadic Atrial Fibrillation

**DOI:** 10.3390/biomedicines14091990

**Published:** 2026-09-03

**Authors:** Zhen-Yu Xu, Dao-Liang Zhang, Xing-Biao Qiu, Chen-Xi Yang, Ying-Jia Xu, Yi-Qing Yang, Ning Li

**Affiliations:** 1 School of Clinical Medicine, Shanghai University of Medicine & Health Sciences, Shanghai 201318, China; zhenyu031128@163.com; 2 Cardiac Arrhythmia Center, Fuwai Hospital, Chinese Academy of Medical Sciences, Shenzhen 518057, China; xkzhangdaoliang@126.com; 3 Department of Cardiology, Shanghai Chest Hospital, Shanghai Jiao Tong University School of Medicine, Shanghai 200030, China; qiuxingbiao@hotmail.com; 4 Department of Cardiology, Shanghai Fifth People’s Hospital, Fudan University, Shanghai 200240, China; cxyang21@m.fudan.edu.cn; 5 Department of Gerontology, Huashan Hospital, Fudan University, Shanghai 200040, China; xuyingjia@5thhospital.com; 6 Cardiovascular Research Laboratory, Shanghai Fifth People’s Hospital, Fudan University, Shanghai 200240, China; 7 Central Laboratory, Shanghai Fifth People’s Hospital, Fudan University, Shanghai 200240, China; 8 Department of Cardiology, The First Affiliated Hospital of Shenzhen University, Shenzhen 518035, China

**Keywords:** atrial fibrillation, human molecular genetics, TBX20, reporter gene analysis

## Abstract

**Background/Objectives**: Atrial fibrillation (AF), the most prevalent form of clinical cardiac arrhythmia globally, is associated with markedly increased morbidity, mortality, and socio-economic expenditure. Accumulating strong evidence highlights genetic abnormalities underpinning its etiopathogenesis. A recent investigation has demonstrated that mutations in the *TBX20* gene, which codes for a T-box transcription factor essential for proper cardiovascular development and structural remodeling, contribute to familial AF. Nevertheless, the mutational prevalence and spectrum of this gene in patients with sporadic AF remain unknown. **Methods**: A cohort of 352 individuals suffering from sporadic AF and a group of 376 healthy subjects without AF history were recruited prospectively. Sanger sequencing examination of *TBX20* was implemented in all research participants. The functional impacts of the discovered *TBX20* variations were quantitatively measured by dual-reporter gene analysis. **Results**: Two novel heterozygous truncating *TBX20* variations, NM_001077653.2: c.725C>A; p.(Ser242*) and NM_001077653.2: c.826A>T; p.(Lys276*), were detected in two of the 352 cases with sporadic AF, respectively, with a mutational prevalence of approximately 0.57%. The two *TBX20* variants were absent from the 752 control chromosomes. Functional measurements revealed that both Ser242* and Lys276* variants lost transactivation on *KCNH2* and *NPPA*, two genes responsible for AF. In addition, each of the two variations abrogated the synergistic transactivation of *NPPA* by TBX20 together with NKX2.5, another gene reported to cause AF. **Conclusions**: The current data indicate haplo-insufficient *TBX20* variations as new genetic defects predisposing to sporadic AF and hence are conducive to improving the prophylaxis and treatment strategies of sporadic AF in a subset of patients.

## 1. Introduction

Atrial fibrillation (AF) represents the most common type of sustained cardiac arrhythmia worldwide, with an estimated global prevalence of 1% in the general population [1,2]. Considering many AF episodes are paroxysmal or asymptomatic, and opportunistic screening with an electrocardiogram (ECG) may miss a diagnosis of AF in a substantial proportion of individuals, the actual prevalence of AF is evidently underestimated [3,4]. In fact, asymptomatic AF reportedly accounts for around 40% of all diagnosed AF cases [5], with ∼11% of AF patients being undiagnosed [6], and the true population prevalence of AF reaches at least 3% when including device-detected asymptomatic AF episodes [7]. Notwithstanding tremendous progress made recently in catheter-based ablation for AF [8], recurrence of AF is observed in up to 40% of post-ablation patients [9]. Therefore, AF has emerged as the third most prevalent cardiovascular disorder, ranking only behind arterial hypertension and ischemic heart disease [10].

Cumulative evidence has demonstrated that in addition to degraded health-correlated quality of life [11,12,13,14] and decreased physical exercise performance [15,16,17], AF also leads to a wide spectrum of other clinically adverse sequelae, encompassing atrial thrombosis [18,19,20], ischemic/thromboembolic cerebral stroke [21,22,23,24], hemorrhagic cerebral stroke [25], cognitive impairment/dementia [26,27,28,29], atrial cardiomyopathy [30,31,32], tachycardia-induced cardiomyopathy [33,34,35], acute myocardial infarction [36,37,38], chronic/congestive heart failure [39,40,41,42], malicious ventricular arrhythmias/cardiac arrest [43,44,45,46], and even premature cardiac death [47,48,49,50,51]. Unmistakably, AF drives substantial morbidity, elevated mortality, and places a staggering economic encumbrance on patients and healthcare systems alike [1,52,53,54,55]. Despite its profound clinical significance, the underlying etiopathogenesis of AF remains unclarified in most affected individuals.

It has been increasingly appreciated that the etiopathogenesis of AF exhibits extraordinary complexity and diversity, and both non-heritable environmental triggers and genetic abnormalities may facilitate the development and persistence of this common tachyarrhythmia [1,2,3,56,57,58,59,60,61]. Mounting research indicates a significant role of non-inheritable etiologic factors in the occurrence of AF, such as unhealthy lifestyles, primary hypertension, gut microbiota dysbiosis, valvular heart disease, acute myocardial infarction, cardiac surgery, obstructive sleep apnea, diabetes mellitus, chronic kidney disease, dilated cardiomyopathy, hyperthyroidism, mitochondrial dysfunction, imbalance of sympathetic and parasympathetic nerve systems, and exposure to environmental toxicants [60,61,62,63,64,65,66,67,68,69,70,71,72]. Yet, accumulating compelling evidence underscores the essential roles of genetic determinants in the initiation and maintenance of AF [1,2,3,56,57,58]. To date, >60 genes causally linked to AF have been identified, the majority of which encode core functional proteins, including *SCN5A*, *KCNH2*, *NPPA/ANP*, *GJC1/Cx45*, *GJA5/Cx40*, *GATA5*, *GATA4*, *TBX5*, and *NKX2.5* [1,2,3,56,57,58,73,74,75,76,77,78,79]. Additionally, single-nucleotide variations at over 520 genomic loci have been significantly associated with AF [2,56,80,81,82]. Nonetheless, owing to the profound genetic heterogeneity inherent to AF, the genetic determinants of AF in most cases remain unknown [1,2,3,56,57,58,73,74,75,76,77,78,79].

Recently, two pathogenic mutations in the *TBX20* gene encoding a core member of the T-box-containing family of transcription factors pivotal for proper cardiovascular development and structural remodeling (NM_001077653.2), c.695A>G (p.His232Arg) and c.862G>C (p.Asp288His), were uncovered to co-segregate with AF in the two families displaying autosomal dominant inheritance, respectively. Functional deciphers revealed that both mutations diminished the transactivation of *KCNH2* and the binding ability to the promoter of *KCNH2*, an already ascertained AF-causative gene [83]. Nevertheless, the mutational prevalence and spectrum of *TBX20* in patients with sporadic AF remain undefined. Consequently, the current investigation aimed to explore the mutational prevalence and spectrum of this gene in patients with sporadic AF and to characterize the functional effect of the identified *TBX20* mutations susceptible to sporadic AF.

## 2. Materials and Methods

### 2.1. Recruitment and Clinical Assessment of Research Cohort

For the present research program, a cohort of 352 unrelated patients affected with sporadic AF, who had a negative family history of AF, alongside a group of 376 unrelated healthy volunteers without an AF history, was enrolled from the Chinese population of Han ethnicity between November 2024 and March 2026. Additionally, once *TBX20* variations were identified in the AF patients, their parents were recruited as study subjects. Clinical diagnosis and categorization of AF were adjudicated by experienced cardiac specialists based on ECG data (including 12-lead ECG tracings, rhythm strip recordings, and available Holter monitor data), in full compliance with the criteria specified elsewhere [1]. The complete set of inclusion/exclusion criteria for the AF patient cohort has been comprehensively detailed in our previously published work [76,78]. Comprehensive clinical data were systematically collected and rigorously evaluated for every enrolled research participant, encompassing detailed family and personal medical histories, standardized physical examination findings, 12-lead ECG recordings, transthoracic echocardiogram reports, and results from a panel of routine clinical laboratory tests. Twenty-four-hour or extended 48 h continuous ambulatory electrocardiographic monitoring was implemented for research participants when clinically indicated. A standardized 12-lead ECG screening was administered to all control subjects to rule out previously undiagnosed AF. The present study protocol received full formal approval from the Ethics Committee of Shanghai Fifth People’s Hospital (approval code: 2022-179; approval date: 23 October 2024), and all research procedures were conducted in strict adherence to the ethical principles laid out in the Declaration of Helsinki. All enrolled research participants provided written informed consent before any study-related procedures or enrollment. Peripheral venous blood samples were collected from each recruited study subject, and high-quality genomic DNA was subsequently extracted using a commercial extraction kit (Cat. No. A1620; Promega, Madison, WI, USA), with DNA concentration and purity measured by spectrophotometry before genetic analysis.

### 2.2. Sequencing Analysis of TBX20 in Research Participants

Using the genomic DNA of each research participant, including the parents of patients carrying the identified *TBX20* variations, as a template, all coding exons alongside flanking splicing donors/acceptors and small parts of 5′ and 3′-untranslated regions of the *TBX20* gene were amplified by polymerase chain reaction (PCR) with the primers as described previously [84]. The amplicons were isolated by electrophoresis on 1.6% agarose gel and extracted with the PureLink™ Quick Gel Extraction Kit (Cat. No. K210025; Invitrogen, Carlsbad, CA, USA). Direct PCR-sequencing assays of the purified amplicons were conducted as narrated elsewhere [84]. As noted elsewhere [3], we retrieved the two public population genetics databases of dbSNP (last updated on 11 March 2025) and gnomAD (version 4.1.1) to ascertain the novelty of all detected *TBX20* variants. Additionally, all identified *TBX20* variants were subsequently classified into distinct pathogenicity categories in strict accordance with the 2015 joint consensus guidelines issued by the American College of Medical Genetics and Genomics and the Association for Molecular Pathology (ACMG/AMP) [85].

### 2.3. Preparation of Gene-Expressing Plasmids

The wild-type human TBX20-pcDNA3.1 expression plasmid and the reporter gene expression plasmid KCNH2-luc, in which the human *KCNH2* promoter drives the expression of firefly luciferase, were constructed as elaborated elsewhere [83]. Using the sequence-verified wild-type human TBX20-pcDNA3.1 expression plasmid as the PCR template, the Ser242*-mutant human TBX20-pcDNA3.1 construct was successfully generated through site-directed mutagenesis, utilizing a commercially available site-directed mutagenesis kit (Cat. No. A13282; Invitrogen, Carlsbad, CA, USA) and a locus-specific pair of primers of 5′-AAGACCACACAGCCTAATTGCTCAACCTGAA-3′ and 5′-TTCAGGTTGAGCAATTAGGCTGTGTGGTCTT-3′. Similarly, the Lys276*-mutant human TBX20-pcDNA3.1 construct was successfully constructed using a locus-specific pair of primers of 5′-CTGATAACGAAGCTGTAAATAGATAGCAATC-3′ and 5′-GATTGCTATCTATTTACAGCTTCGTTATCAG-3′. The wild-type human NKX2.5-pEFSA expression plasmid was described elsewhere [86]. The reporter gene expression plasmid NPPA-luc, in which the human *NPPA* promoter induces the expression of firefly luciferase, was constructed as described previously [87]. All the above-mentioned expression plasmids were confirmed by DNA sequencing analysis.

### 2.4. Cellular Transfection with Expression Plasmids Followed by Dual-Reporter Assay

As described elsewhere [83], HeLa cells (Cat. No. SCSP-504; National Collection of Authenticated Cell Cultures, Shanghai, China) were cultivated to be ~80% confluent and transfected with the indicated amounts of expression plasmids utilizing Lipofectamine^®^ LTX & PLUS™ Reagent (Cat. No. 15338-100 & Cat. No. 11514-015; Invitrogen, Carlsbad, CA, USA) as per the manufacturer’s manual. Specifically, HeLa cells were transfected with 0.1 μg of empty pcDNA3.1 plasmid (Cat. No. V79020; Invitrogen, Carlsbad, CA, USA) or 0.1 μg of wild-type TBX20-pcDNA3.1 plasmid or 0.1 μg of mutant TBX20-pcDNA3.1 plasmid or 0.05 μg of wild-type TBX20-pcDNA3.1 plasmid + 0.05 μg of empty pcDNA3.1 plasmid or 0.05 μg of wild-type TBX20-pcDNA3.1 plasmid + 0.05 μg of mutant TBX20-pcDNA3.1 plasmid, in combination with 1.0 μg of the KCNH2-luc plasmid and 0.04 μg of the pRL-TK plasmid expressing renilla luciferase (Cat. No. E2241; Promega, Madison, WI, USA). As described previously [83,88], the HeLa and COS7 cells were collected and lysed, and the fluorescence intensities of firefly and renilla luciferases in the cellular lysates were sequentially measured and normalized using the Dual-Glo^®^ Luciferase Assay System (Cat. No. E2940; Promega, Madison, WI, USA), performed using the GloMax^®^ Discover System in accordance with the manufacturer’s instructions. The transcriptional activities of the *KCNH2* and *NPPA* promoters were expressed as the ratios of firefly luminescence intensities to renilla luminescence intensities. For each plasmid used, three parallel functional experiments were performed in triplicate, and the mean ± standard deviation (SD) was calculated from three experimental results, which was used for comparison between/among groups.

### 2.5. Statistics

As described elsewhere [89,90], continuous data, such as ages and promoter activity measurements, are presented as mean ± SD when distributed normally. Statistical analyses of continuous data were performed using the unpaired two-tailed Student’s *t*-test for pairwise comparisons, or one-way analysis of variance (ANOVA) followed by the Tukey–Kramer honestly significant difference (HSD) post hoc test for multi-group comparisons. Categorical parameters, such as sex and stroke history, are expressed as number (*n*) alongside percentage (%) and compared pairwise with Pearson’s chi-square test or Fisher’s exact test. A two-tailed *p*-value < 0.05 was defined as the threshold for statistical significance.

## 3. Results

### 3.1. Baseline Clinical Characteristic Data of the Study Participants

In the present study, a cohort of 352 cases with sporadic AF underwent a comprehensive clinical investigation in contrast to a group of 376 healthy volunteers without AF, along with 4 healthy individuals as the parents of two unrelated AF cases harboring two identified TBX20 variations. All study participants were recruited from the Han-race Chinese population in the same geographic region. None of the study subjects had known environmental etiologic factors for AF, such as obesity, essential hypertension, obstructive sleep apnea, hyperthyroidism, chronic renal disease, valvular heart disease, coronary heart disease, and prior cardiac surgery. The patient group exhibited higher rates of ischemic cerebral stroke, greater proportions of pacemaker implantation, and larger left atrial diameters compared to the control group. No significant differences were observed in ethnicity, gender, or age between the two groups. The demographic and baseline clinical characteristics of the 352 patients with sporadic AF and 376 healthy subjects are summarized in Table 1.

### 3.2. Discovery of Two New TBX20 Mutations Enhancing the Susceptibility to AF

**Figure 1:**
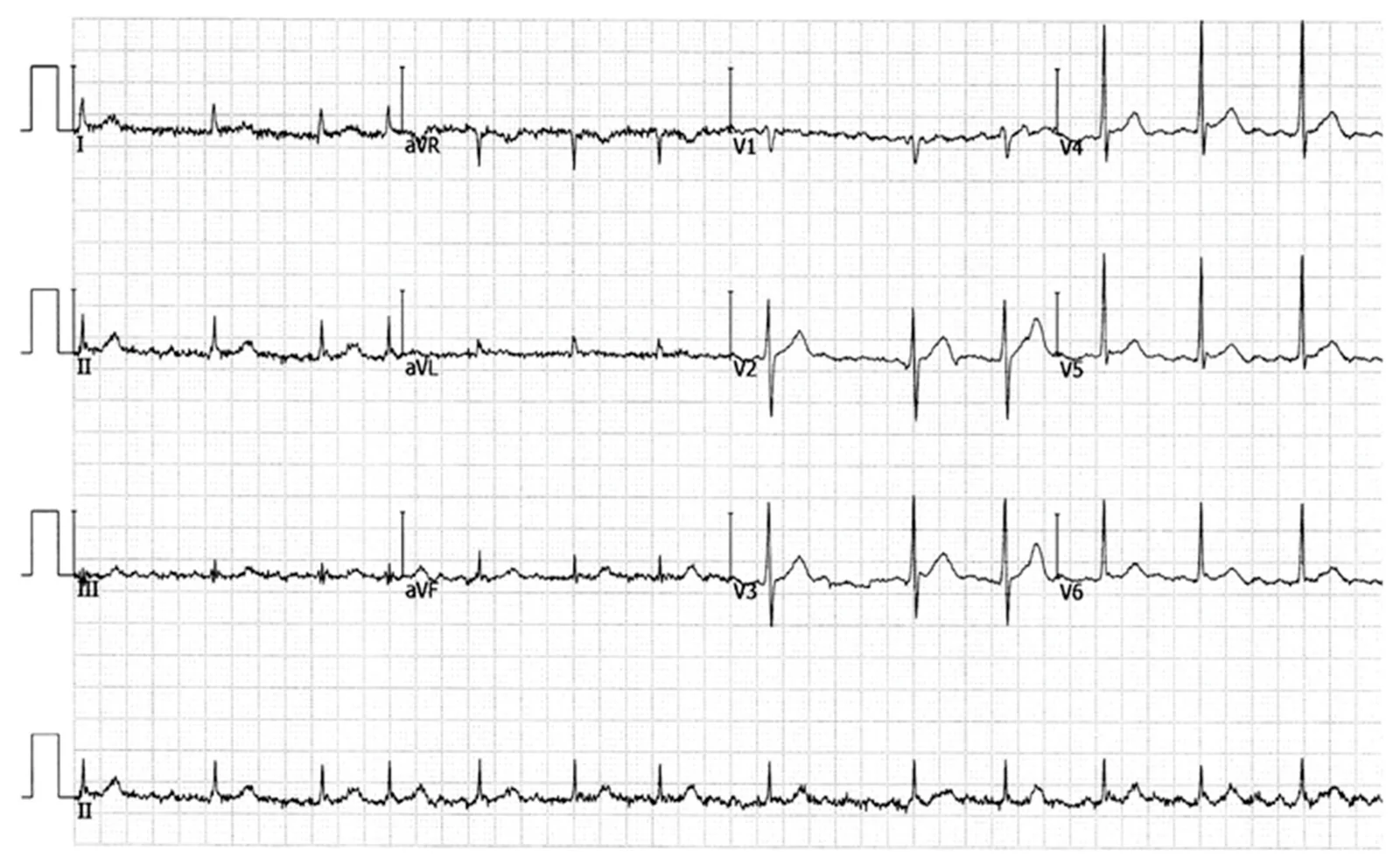
A 12-lead electrocardiogram (ECG) recorded from the male patient suffering from sporadic atrial fibrillation (AF) carrying the *TBX20* c.725C>A mutation. This representative standard ECG illustrates AF.

Via Sanger sequencing assay of *TBX20* in 352 unrelated cases affected with sporadic AF, two heterozygous truncating *TBX20* mutations, NM_001077653.2: c.725C>A; p.(Ser242*) and NM_001077653.2: c.826A>T; p.(Lys276*), were detected in two unrelated cases, respectively, with a total mutational prevalence of roughly 0.57%. Specifically, the *TBX20* c.725C>A mutation was observed in a 41-year-old male patient with idiopathic AF without a family history of AF. This *TBX20* mutation was not detected by the Sanger sequencing analysis in his healthy parents, indicating a de novo mutation. He was initially diagnosed with paroxysmal AF at the age of 32 years; at the time of the current study recruitment, he was diagnosed with long-standing persistent AF and subsequently underwent a successful catheter-based radiofrequency ablation of AF, whose one representative 12-lead ECG recorded prior to radiofrequency ablation documenting AF is provided as Figure 1.

**Figure 2:**
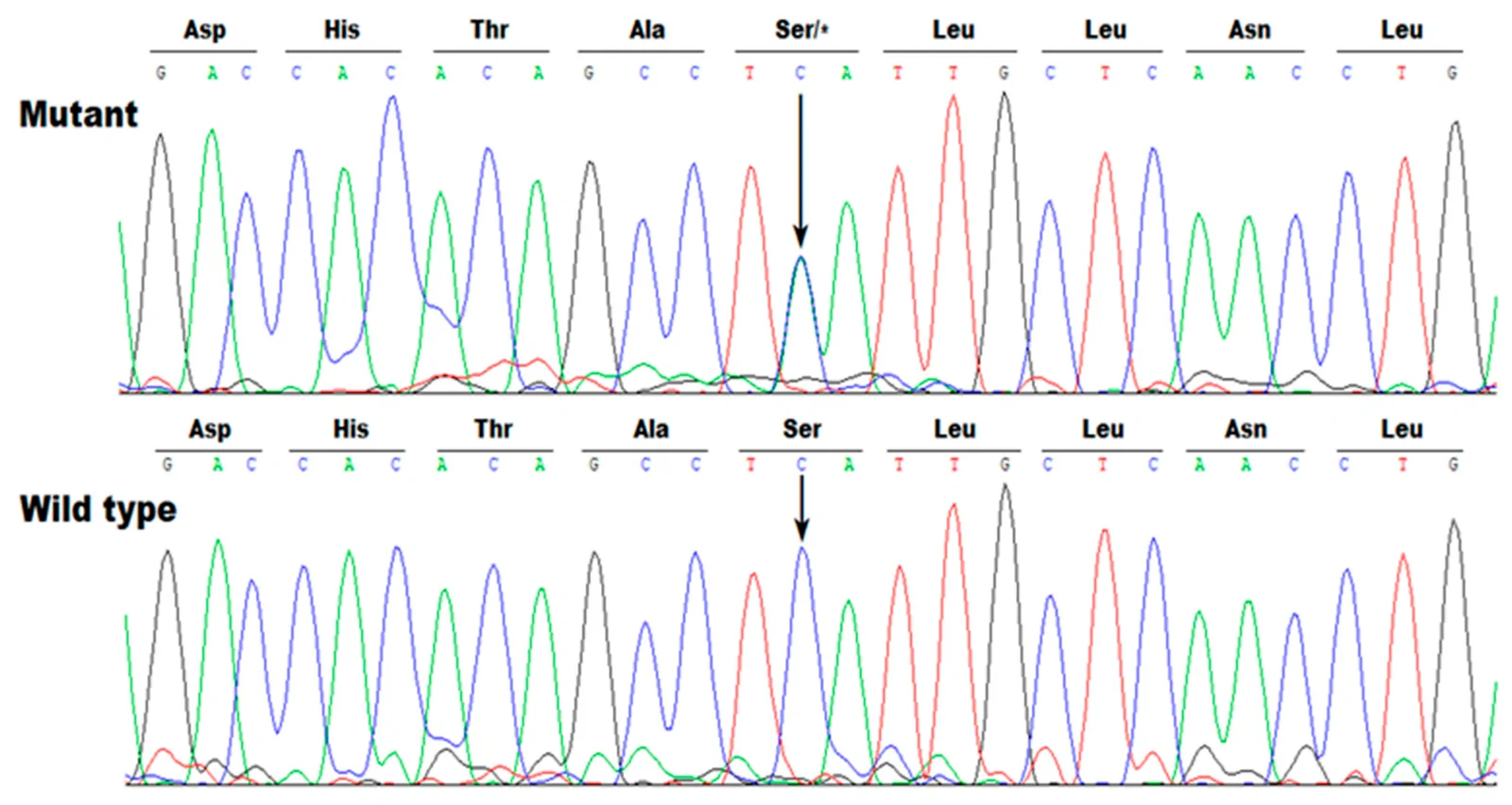
A truncating *TBX20* mutation contributing to sporadic atrial fibrillation (AF). The sequence electropherogram traces show the heterozygous *TBX20* c.725C>A mutation observed in the male case suffering from sporadic AF (Mutant), along with its wild-type control detected in a non-AF healthy subject (Wild type).

The sequencing chromatograms illustrating the heterozygous *TBX20* c.725C>A mutation alongside its wild-type control are displayed in Figure 2.

**Figure 3:**
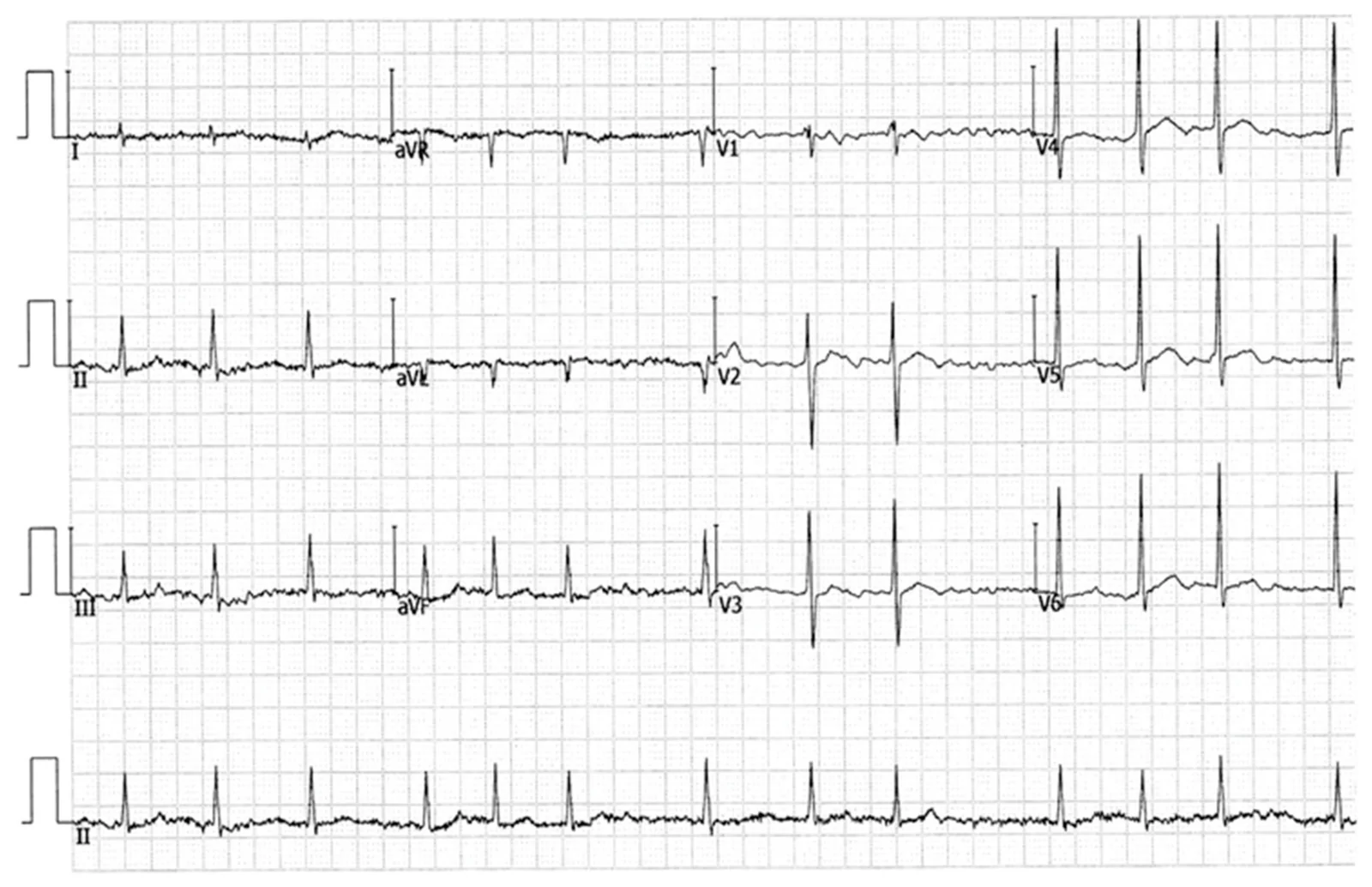
A 12-lead electrocardiogram (ECG) recorded from the female case affected with sporadic atrial fibrillation (AF) carrying the *TBX20* c.826A>T mutation. This representative ECG documents AF.

Additionally, the *TBX20* c.826A>T mutation was discovered in a 48-year-old female case affected with idiopathic AF without a family history of AF. This *TBX20* mutation was not observed by the Sanger sequencing assay in her healthy parents, indicating a de novo mutation. She was first diagnosed with persistent AF at the age of 43 years; at the time of the current study enrollment, she was diagnosed with long-standing persistent AF and then underwent a successful catheter-based radiofrequency ablation of AF, whose one representative 12-lead ECG recorded prior to radiofrequency ablation documenting AF is given as Figure 3.

**Figure 4:**
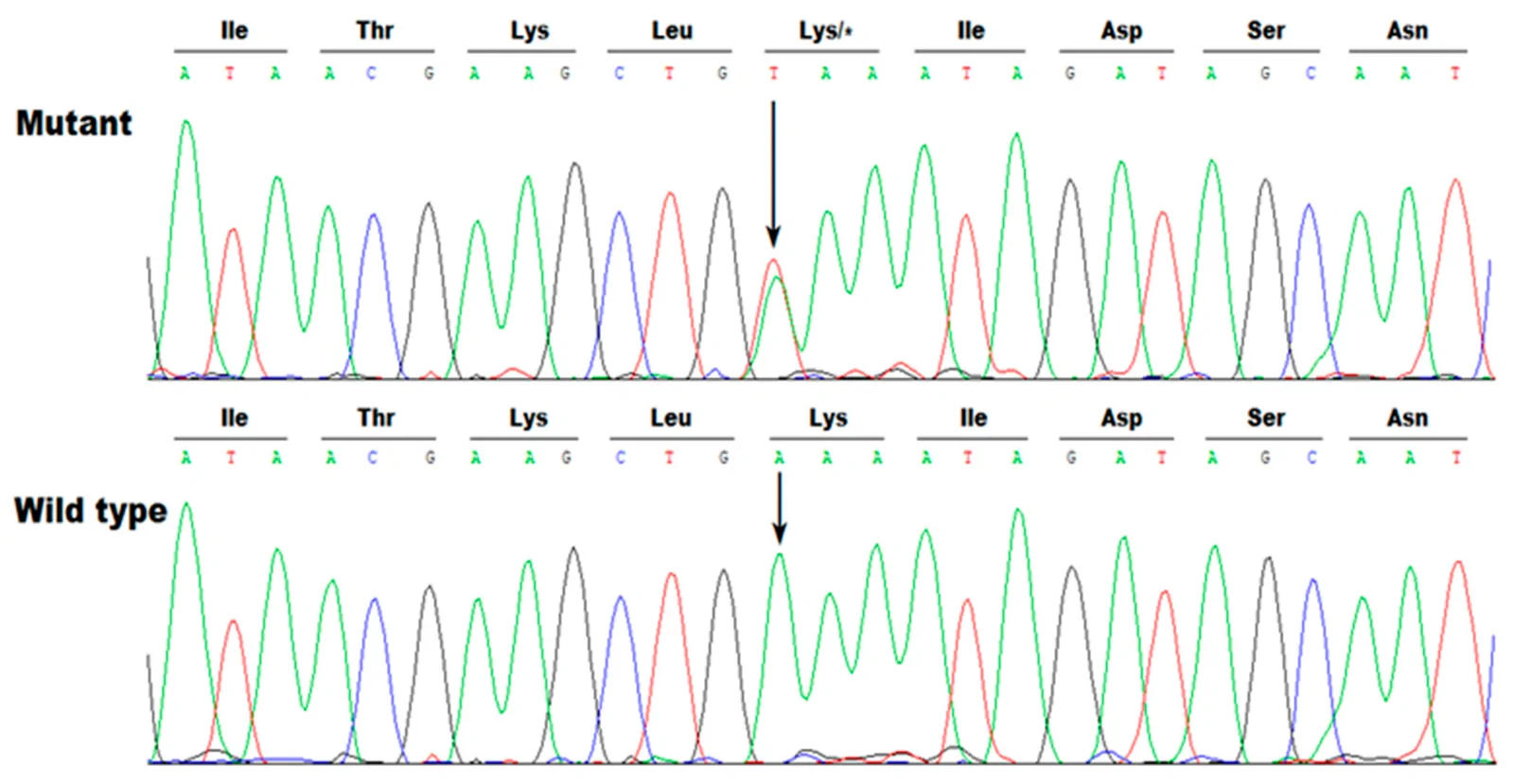
A nonsense *TBX20* mutation predisposing to sporadic atrial fibrillation (AF). The sequence electropherogram traces show the heterozygous *TBX20* c.826A>T mutation discovered in the female patient affected with sporadic AF (Mutant), alongside its wild-type control detected in a non-AF healthy individual (Wild type).

The sequencing electropherograms illustrating the heterozygous *TBX20* c.826A>T mutation alongside its wild-type control are exhibited in Figure 4.

The two heterozygous, truncating *TBX20* variations implicated with sporadic AF, NM_001077653.2: c.725C>A; p.(Ser242*) and NM_001077653.2: c.826A>T; p.(Lys276*), were neither present in the 376 non-AF healthy folks nor released from the population genetics databases of gnomAD (version 4.1.1; accessed on 16 July 2026) and dbSNP (updated 15 May 2025; accessed on 16 July 2026), indicating the novelty of the two truncating *TBX20* variations.

### 3.3. Failure of Ser242*- or Lys276*-Mutant TBX20 to Transactivate KCNH2

**Figure 5:**
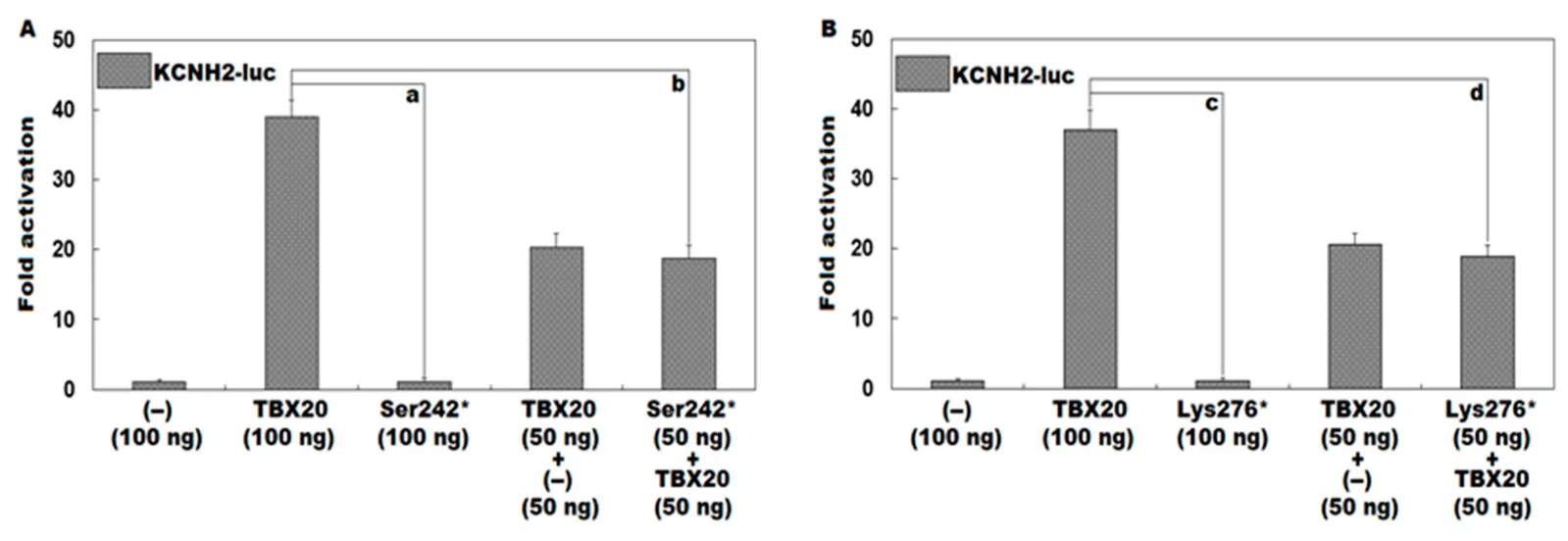
Failure of Ser242*- or Lys276*-mutant TBX20 to transactivate *KCNH2*. In HeLa cells cultured in vitro, dual-luciferase activity measurements revealed that both the Ser242*-mutant TBX20 (**A**) and the Lys276*-mutant TBX20 (**B**) failed to transactivate *KCNH2*. Here, “a” and “c” represent *p* < 0.0001; “b” and “d” signify *p* < 0.001, when compared to wild-type TBX20 (100 ng). An error bar represents SD.

As Figure 5A displayed, in the cultivated HeLa cells transfected with indicated expression plasmids, encompassing empty pcDNA3.1 plasmid (−), wild-type human TBX20-pcDNA3.1 plasmid (TBX20), and Ser242*-mutant human TBX20-pcDNA3.1 plasmid (Ser242*), singly or together, TBX20 and Ser242* transactivated *KCNH2* by ~39-fold and ~1-fold, respectively (using an unpaired Student’s *t*-test, TBX20 versus Ser242*: *t* = 26.6330; *p* < 0.0001). When TBX20 and Ser242* were co-expressed, the elicited transactivation of *KCNH2* was ~19-fold (using an unpaired Student’s *t*-test, TBX20 versus Ser242* + TBX20: *t* = 11.5659; *p* = 0.0003). In addition, similar statistical significance was observed from multiple comparisons (F = 280.4163, *p* = 3.209 × 10^−10^). Specifically, using a Tukey–Kramer-HSD post hoc test, for TBX20 versus Ser242*, *t* = 37.7733; *p* < 0.0001; for TBX20 versus Ser242* + TBX20, *t* = 20.13; *p* < 0.0001; for TBX20 + (−) versus TBX20 + Ser242*, *t* = 1.4867; *p* = 0.7954. Similarly, as Figure 5B shows, TBX20 and Lys276* transactivated *KCNH2* by ~37-fold and ~1-fold, respectively (using an unpaired Student’s *t*-test, TBX20 versus Lys276*: *t* = 21.1207; *p* < 0.0001). When TBX20 and Lys276* were co-expressed, the elicited transactivation of *KCNH2* was ~19-fold (using an unpaired Student’s *t*-test, TBX20 versus Lys276* + TBX20: *t* = 9.4597; *p* = 0.0007). In addition, similar statistical significance was observed from multiple comparisons (F = 245.6054, *p* = 6.18 × 10^−10^). Specifically, using a Tukey–Kramer-HSD post hoc test, for TBX20 versus Lys276*, *t* = 35.8; *p* < 0.0001; for TBX20 versus Lys276* + TBX20, *t* = 18.0333; *p* < 0.0001; for TBX20 + (−) versus TBX20 + Lys276*, *t* = 1.68; *p* = 0.7339.

### 3.4. Inability of Ser242*-or Lys276*-Mutant TBX20 to Transactivate NPPA Alone or Synergistically with NKX2.5

**Figure 6:**
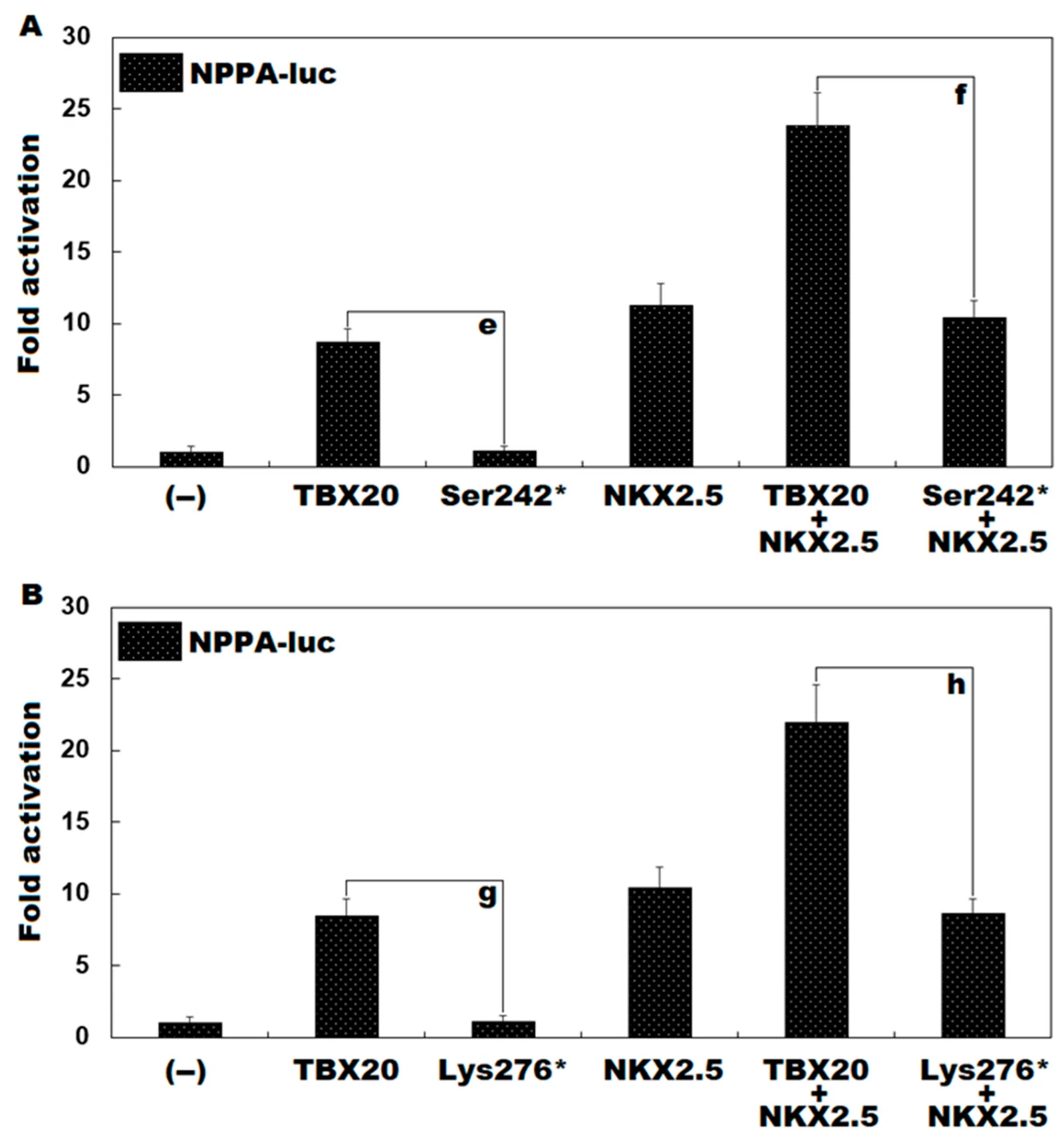
Synergistic activation of *NPPA* between TBX20 and NKX2.5 nullified by the Ser242* or Lys276* mutation. In COS7 cells cultivated in vitro, biochemical assays with dual-reporter genes demonstrated that the synergistic activation of *NPPA* by TBX20 and NKX2.5 was abolished by the Ser242* mutation (**A**) or the Lys276* mutation (**B**). Here, “e”, “f”, and “g” indicate *p* < 0.001; “h” denotes *p* < 0.005, in comparison with the corresponding wild-type counterparts. An error bar represents SD.

As manifested in Figure 6A, in the cultivated COS7 cells transfected with indicated plasmids, including empty pcDNA3.1 plasmid (−), wild-type human TBX20-pcDNA3.1 plasmid (TBX20), Ser242*-mutant human TBX20-pcDNA3.1 plasmid (Ser242*), and NKX2.5-pEFSA plasmid (NKX2.5), singly or in combination, TBX20 and Ser242* transactivated *NPPA* by ~9-fold and ~1-fold, respectively (TBX20 versus Ser242*: *t* = 13.2126; *p* = 0.0002). In combination of NKX2.5, TBX20 and Ser242* transactivated *NPPA* by ~24-fold and ~10-fold, respectively (TBX20 + NKX2.5 versus Ser242* + NKX2.5: *t* = 9.1171; *p* = 0.0008). Additionally, similar statistical results were obtained from multiple comparisons (F = 127.148, *p* = 5.787 × 10^−10^). Specifically, for TBX20 versus Ser242*, *t* = 7.6; *p* = 0.0001; for TBX20 + NKX2.5 versus Ser242* + NKX2.5, *t* = 13.3933; *p* < 0.0001. Analogously, as delineated in Figure 6B, TBX20 and Lys276* transactivated *NPPA* by ~8-fold and ~1-fold, respectively (TBX20 versus Lys276*: *t* = 10.5529; *p* = 0.0005). In combination of NKX2.5, TBX20 and Lys276* transactivated *NPPA* by ~22-fold and ~9-fold, respectively (TBX20 + NKX2.5 versus Lys276* + NKX2.5: *t* = 8.2338; *p* = 0.0012). Additionally, similar statistical results were obtained from multiple comparisons (F = 92.8586, *p* = 3.63 × 10^−9^). Specifically, for TBX20 versus Lys276*, *t* = 7.36; *p* = 0.0003; for TBX20 + NKX2.5 versus Lys276* + NKX2.5, *t* = 13.3033; *p* < 0.0001.

## 4. Discussion

In the current investigation, two heterozygous, truncating variations in *TBX20* were uncovered in two of 352 unrelated individuals with sporadic AF, respectively, including c.725C>A leading to p.Ser242* (NM_001077653.2) and c.826A>T leading to p.Lys276* (NM_001077653.2). Each *TBX20* variant occurred in one of the 352 unrelated cases, giving a total mutational frequency of approximately 0.57%. Neither TBX20 variation was observed in the 752 human control chromosomes nor reported in such databases as gnomAD and dbSNP. The two de novo truncating *TBX20* variations were both classified as pathogenic mutations based on the ACMG/AMP criteria for pathogenicity: (i) 1 very strong (PVS1, a nonsense variant in a gene where loss of function is a known mechanism of disease) and (a) ≥1 strong (PS2, a de novo variant, and PS3, well-established in vitro functional studies supportive of a damaging effect on the gene or gene product) [85]. Experimental functional analyses demonstrated that both truncating *TBX20* variants (Ser242* and Lys276*) eliminated TBX20-driven transcriptional activation of *KCNH2* and *NPPA*, two genes implicated in AF [73,91,92,93,94,95]. Furthermore, each variant also disrupted the cooperative/synergistic activation of *NPPA* produced by the combined action of TBX20 and NKX2.5, a transcription factor likewise associated with AF [96,97,98]. Notably, the non-significant difference between “TBX20 + empty vector” and “Ser242* + TBX20” (*t* = 1.4867; *p* = 0.7954) or “Lys276* + TBX20” (*t* = 1.68; *p* = 0.7339) suggests a haploinsufficiency mechanism rather than dominant-negative action. Consequently, genetically impaired *TBX20* may enhance the susceptibility to sporadic AF by lowering the expression of its downstream target genes, including *KCNH2* and *NPPA*.

The T-box (TBX) family of transcription factors, such as TBX20, TBX5, TBX3, and TBX1, is considered essential for normal heart development [99]. They participate in key processes throughout embryogenesis in vertebrates, including establishing cardiac cell identity early on, forming the valves and septum (valvuloseptal development), specifying the different heart chambers, and helping generate and diversify the cardiac conduction system [99]. The TBX transcription factors contain a functionally important TBX domain, which recognizes and binds specific DNA sequences known as T-half sites (5′-AGGTGTGA-3′) located in the promoters and enhancers of downstream target genes, and hence can either activate transcription (transactivation) or suppress it (transcriptional repression), enabling highly coordinated temporal–spatial control of gene expression during cardiac morphogenesis [83,99]. In addition, the TBX domain is also required for the interaction between TBX transcription factors and other transcriptional regulators/transcriptional factor partners [100]. In humans, the *TBX20* gene is mapped on chromosome 7p14.2 and encodes a protein made up of 447 amino acids [83]. Earlier experimental studies have demonstrated that TBX20 is highly expressed in both the developing heart and the adult heart [101], where it functions as a transcriptional activator for many heart-expressed target genes, encompassing *KCNH2*, *NPPA*/*ANP*, *GJC1*/*Cx45*, and *GJA5*/*Cx40*, alone or synergistically with cooperating partners such as NKX2.5, TBX5, GATA5, and GATA4 [83,102,103]. In addition to *KCNH2* [91,92,93], *NPPA* [73,94,95], and *NKX2.5* [96,97,98], pathogenic mutations in such genes as *GJC1* [104], *GJA5* [105,106,107], *GATA5* [108,109], *GATA4* [110,111,112], and *TBX5* [113,114,115] have been reported to give rise to AF. In our previous study, two novel loss-of-function mutations in *TBX20* were uncovered to contribute to familial AF [83]; in our present investigation, two novel loss-of-function mutations in *TBX20* were associated with enhanced susceptibility to sporadic AF. These findings strongly suggest that impaired *TBX20* function increases the vulnerability to both familial and sporadic AF, most likely by reducing the expression of downstream target genes.

The fact that genetically compromised *TBX20* enhances the susceptibility to AF may be at least in part attributable to structural remodeling and electrophysiological anomalies of the heart [83,100,101,102,103,116]. Prior research has highlighted the crucial roles of *TBX20* in multiple aspects of heart biology, including embryonic cardiac organogenesis, postnatal structural adaptation to maintain internal homeostasis, and cardiac electrophysiology [101]. The cardiac conduction system comprises specialized cells and structures of the heart, encompassing the sinoatrial node, atrioventricular node, atrioventricular bundle, the left and right bundle branches, and the network of Purkinje fibers, which precisely routes the electrical impulse in a spatiotemporal way, eliciting a synchronized heart rhythm and coordinated cardiac contraction [101]. In addition, the heart’s contractile (working) cardiomyocytes are also important for spreading electrical impulses through the myocardium [101]. Aggregating evidence indicates that cardiac conduction system malfunctions may lead to AF and life-threatening ventricular arrhythmias and raise the risk of sudden unexpected death in affected patients [117,118,119,120,121]. Whole-genome association studies have linked variants in/near *TBX20* to a prolonged QRS duration, suggesting that *TBX20* may participate in forming and/or maintaining the cardiac conduction system and in regulating electrical signaling through the myocardium [117,122,123]. TBX20 probably helps organize and preserve the spatiotemporal control required for both the formation and proper functioning of the cardiac conduction system, acting through interconnected, multigene regulatory pathways [101]. Recent studies revealed that mice in which *Tbx20* was selectively deleted from adult cardiomyocytes developed enlarged hearts, lost contractile performance, showed reduced conduction speed, and developed serious arrhythmia [124,125]. Chromatin immunoprecipitation and enhancer-mapping experiments further verified that TBX20 directly controls a broad set of target genes involved in regulating heart rhythm [126], and deleterious variations in many of these downstream genes were found to underlie inherited ion-channel disorders in humans [124,126,127,128]. Notably, two recent studies demonstrated that TBX20 can transactivate the expression of KCNH2 [83,129]. The *KCNH2* gene encodes Kv11.1, also known as hERG, which forms the pore-forming α subunit, constituting a rapidly activating potassium channel together with the auxiliary β subunit encoded by KCNE2, which produces potassium channel currents that are major contributors to myocardial repolarization [129,130]. Further research found that the TBX20 Arg311Cys variant identified in families suffering from long QT syndrome nullified TBX20’s transactivation activity, leading to reduced hERG expression and a smaller inward-rectifier current, responsible for prolonged cardiac action potential [129]. It is widely accepted that triggered, or ectopic, focal electric activity and micro-reentry are the two main mechanisms of AF, and triggered activity can arise from early afterdepolarizations, which are promoted by a prolonged action potential [131,132]. Moreover, it has been reported that TBX20 can also regulate the expression of RYR2, KCND3, KCND2, CACNA1C, CACNA1A, and CAMK2D, underlining the critical role of TBX20 in maintaining the proper cardiac electrophysiology [101,124,131,132]. Taken together, these findings strongly suggest that genetically defective *TBX20* predisposes to sporadic AF in humans.

Previously in humans, a diverse panel of *TBX20* loss-of-function and gain-of-function mutations has been linked to a wide spectrum of clinically heterogeneous congenital heart defects (CHDs), spanning ventricular septal defects (VSDs), atrial septal defects (ASDs), double-outlet right ventricle (DORV), tetralogy of Fallot (TOF), patent ductus arteriosus (PDA), pentalogy of Fallot, persistent truncus arteriosus (PTA), complete common atrioventricular canal (CAVC), coarctation of the aorta (CoA), anomalous pulmonary venous connection (APVC), and cardiac valve deformations such as bicuspid aortic valve (BAV) [84,88,133,134,135]. Additionally, multiple *TBX20* loss-of-function mutations have also been linked to dilated cardiomyopathy (DCM) [116,136,137,138] and familial AF in humans [83]. These cumulative clinical and genetic findings, together with the present observational results, rigorously establish the non-redundant, dose-dependent criticality of *TBX20* in orchestrating early human cardiogenesis and postnatal cardiac structural and electrophysiological homeostasis, providing supportive genetic evidence that pathogenic *TBX20* variants confer a strong heritable predisposition to distinct cardiac pathologies, including CHDs, DCM, and AF.

There are limitations to the present studies, especially to the luciferase assays performed in HeLa and COS7 cells, which are non-cardiac cell lines. HeLa cells are derived from a cervical epithelial cancer line, and COS7 cells are a fibroblast-like cell line derived from African green monkey kidney tissue, without myocardial transcriptional architecture, cardiomyocyte-specific co-factors, or electrophysiological phenotype [3]. Provided that TBX20 is a transcription factor crucial for cardiac development with its activity highly context-dependent—needing interaction with transcriptionally cooperative partners such as GATA4, NKX2-5, and TBX5 [133], as well as cardiac chromatin accessibility—the electrophysiological significance of transactivation data from non-myocardial cell lines is questionable, mainly because functional assays in HeLa and COS7 cells rather than cardiomyocyte-derived cell models may not fully recapitulate the real transcriptional regulatory network of human atrial tissue [3]. Functional explorations in cardiomyocyte-derived cell lines, such as HL-1 cells, neonatal cardiomyocytes, AC16 cells, induced pluripotent stem cell-derived cardiomyocytes (iPSC-CMs), and ideally human atrial cardiomyocytes, will substantially strengthen the experimental findings [3]. Nevertheless, non-cardiac cell lines, such as HeLa cells, were commonly utilized to assess the functional effects of cardiac transcription factor mutations, including GATA4 and HAND2 mutations, which minimized the adverse functional impact of various endogenous transcription factors [3,139]. Secondly, the mechanistic claim rests solely on luciferase reporter activity in HeLa/COS7 cells, without orthogonal evidence such as mutant protein expression/stability (Western blot), DNA-binding assays (EMSA/ChIP), or electrophysiological validation (patch-clamp or iPSC-CM). Finally, Table 1 compares 15 demographic and baseline variables without correction for multiple comparisons; this is another limitation, especially since stroke history and left atrial diameter differences may simply reflect AF consequences rather than *TBX20* genotype effects.

## 5. Conclusions

This study is the first to causally link loss-of-function variations in *TBX20* to sporadic AF in humans, supporting an association between genetically defective *TBX20* and enhanced susceptibility to sporadic AF. These findings yield unprecedented mechanistic insights into the molecular etiopathogenesis underpinning sporadic AF and unveil a promising molecular target for the genetic counseling and prognostic risk evaluation of mutation carriers.

## Figures and Tables

**Table 1:** Demographic and baseline phenotypical features of the study participants.

Individual Parameter	AF Patient Group (*n* = 352)	Healthy Control Group (*n* = 376)	Two-Tailed *p*-Value
Age at recruitment (years)	51.7 ± 6.3	52.1 ± 7.0	0.4191
Sex (female/male)	159/193	170/206	0.9909
History of cerebral stroke (%)	13 (4)	0 (0)	0.0001 *
History of pacemaker implantation (%)	10 (3)	0 (0)	0.0013 *
Body mass index (kg/m^2^)	24.0 ± 3.7	23.8 ± 3.6	0.4601
Diastolic blood pressure (mmHg)	83.3 ± 6.4	82.7 ± 7.1	0.2325
Systolic blood pressure (mmHg)	125.2 ± 10.8	126.0 ± 11.2	0.3275
Serum total cholesterol (mmol/L)	3.9 ± 0.7	3.9 ± 0.8	>0.9999
Serum triglyceride (mmol/L)	1.3 ± 0.4	1.3 ± 0.5	>0.9999
Fasting venous plasma glucose (mmol/L)	4.8 ± 0.8	4.8 ± 0.7	>0.9999
Venous plasma HbA1c (%)	3.7 ± 0.6	3.7 ± 0.5	>0.9999
Venous plasma creatinine (µmol/L)	64.7 ± 12.0	65.2 ± 11.4	0.5644
Left ventricular ejection fraction (%)	62.6 ± 7.1	63.1 ± 8.9	0.4043
Left atrial diameter (mm)	38.7 ± 6.4	35.4 ± 5.3	<0.0001 *
Resting ventricular rate (beats/min)	77.4 ± 13.2	76.5 ± 12.6	0.3469

AF, atrial fibrillation; HbA1c, glycated hemoglobin A1c. * indicates significant difference.

## Data Availability

All data generated in the current study are included in this paper.

## Abbreviations

The following abbreviations are used in this manuscript:

AF	Atrial Fibrillation
TBX20	T-box transcription factor 20
ECG	Electrocardiogram
SCN5A	Sodium voltage-gated channel alpha subunit 5
SCN10A	Sodium voltage-gated channel alpha subunit 10
SCN4B	Sodium voltage-gated channel beta subunit 4
SCN1B	Sodium voltage-gated channel beta subunit 1
KCNQ1	Potassium voltage-gated channel subfamily Q member 1
KCNH2	Potassium voltage-gated channel subfamily H member 2
KCNE2	Potassium voltage-gated channel subfamily E regulatory subunit 2
KCNE1	Potassium voltage-gated channel subfamily E regulatory subunit 1
Cx40	Connexin 40
GJA5	Gap junction protein alpha 5
Cx43	Connexin 43
GJA1	Gap junction protein alpha 1
NPPA	Natriuretic peptide precursor A
NKX2.5	NK2 homeobox 5
PITX2	Paired like homeodomain 2
ZFHX3	Zinc finger homeobox 3
KCNN3	Potassium calcium-activated channel subfamily N member 3
PCR	Polymerase chain reaction
ACMG	American College of Medical Genetics
AMP	Association for Molecular Pathology
dbSNP	Database of Single Nucleotide Polymorphism
gnomAD	Genome Aggregation Database
KCNH2-luc	KCNH2-luciferase
NPPA-luc	NPPA-luciferase
SD	Standard deviation
ANOVA	One-way analysis of variance
HSD	Honestly significant difference
HbA1c	Glycated hemoglobin A1c
TBX5	T-box transcription factor 5
GATA5	GATA binding protein 5
GATA4	GATA binding protein 4
RYR2	Ryanodine receptor 2
KCND3	Potassium voltage-gated channel subfamily D member 3
KCND2	Potassium voltage-gated channel subfamily D member 2
CACNA1C	Calcium voltage-gated channel auxiliary subunit alpha1C
CACNA1A	Calcium voltage-gated channel auxiliary subunit alpha1A
CAMK2D	Calcium/calmodulin dependent protein kinase II delta
CHD	Congenital heart defect
VSD	Ventricular septal defect
ASD	Atrial septal defect
DORV	Double-outlet right ventricle
TOF	Tetralogy of Fallot
PDA	Patent ductus arteriosus
PTA	Persistent truncus arteriosus
CAVC	Complete common atrioventricular canal
CoA	Coarctation of the aorta
APVC	Anomalous pulmonary venous connection
BAV	Bicuspid aortic valve
DCM	Dilated cardiomyopathy
iPSC-CMs	Induced pluripotent stem cell-derived cardiomyocytes
EMSA	Electrophoretic mobility shift assay
ChIP	Chromatin immunoprecipitation
